# The prevalence, promotion and pricing of three IVF add-ons on fertility clinic websites

**DOI:** 10.1016/j.rbmo.2020.07.021

**Published:** 2020-11

**Authors:** Lucy van de Wiel, Jack Wilkinson, Pantelitsa Athanasiou, Joyce Harper

**Affiliations:** a Department of Sociology, University of Cambridge, Cambridge, UK; b Centre for Biostatistics, Faculty of Biology, Medicine and Health, Manchester Academic Health Science Centre, University of Manchester Manchester, UK; c Institute for Women's Health, University College London, London, UK

**Keywords:** Add-ons, Assisted hatching, IVF, PGT-A, Time-lapse embryo imaging

## Abstract

**Research question:**

How are IVF clinic websites advertising three common IVF add-ons: assisted hatching, time-lapse embryo imaging and preimplantation genetic testing for aneuploidies (PGT-A)?

**Design:**

The Human Fertilisation and Embryology Authority ‘Choose a fertility clinic‘ website service was used to identify IVF clinics and their websites. Assisted hatching, time-lapse embryo imaging and PGT-A were examined to determine which websites advertised them, what price they charged and what claims they made in relation to the add-ons.

**Results:**

Eighty-seven eligible clinics were identified, with 72 unique websites; 37 (43%) clinics were part of one of nine groups of IVF clinics, of sizes ranging from two to eight clinics in the UK. Time-lapse imaging (TLI) was the most frequently advertised of the three add-ons (67% of clinics), followed by PGT-A (47%) and assisted hatching (28%). Very few websites stated that the effectiveness of the add-on was in doubt or unclear (four, two and one websites for TLI, PGT-A and assisted hatching, respectively), and none raised the possibility that an add-on might have negative effects. Claims of efficacy were often based on upstream outcomes (e.g. implantation, pregnancy). Some claims that PGT-A and TLI improved live birth rates were found. There was substantial variation in pricing.

**Conclusions:**

IVF clinic websites provide valuable information for patients seeking fertility treatment so it is key that the information is accurate and complete. There is a need for transparent information on interventions, including uncertainties and risks, to be made available by IVF clinics to support well-informed treatment decisions. The selected add-ons are widely advertised, and there is wide variation in pricing.

## Introduction

Since the birth of Louise Brown in 1978 (Steptoe and Edwards, 1978), millions of children have been born following IVF, and assisted reproduction has become a relatively privatized and lucrative medical industry. In the last decade, a wide variety of adjunct treatments or tests have been introduced in fertility clinics, while major biotechnology and pharmaceutical companies are investing in these so-called ‘add-ons.’ Adjuncts are defined as any technique that is a variation of, or add-on to, the ‘normal’ IVF cycle. This includes laboratory, clinical and complementary treatments. Although IVF clinics seek to help patients increase their chances of having a baby by offering add-ons, the evidence base for their effectiveness is variable but generally limited. Both the Human Fertilisation and Embryology Authority (HFEA), which is the UK regulator of IVF, and recent scientific reviews have found that there is limited high-quality evidence to support the use of add-ons in routine practice (Armstrong et al., 2019; Farquhar, 2019; Harper et al., 2017; HFEA 2018).

Add-on treatments have generated much discussion in the fertility field throughout the last decade. Concerns have been raised about the commercial drivers behind their introduction and the possible tensions between direct-to-consumer advertising and the scope for patients to make informed decisions (Day 2016; Harper et al., 2017; Rutherford 2017). Beyond efficacy, add-ons such as time-lapse embryo imaging have also raised questions about the patenting of embryo development and the creation of technological lock-ins and power asymmetries through standardization, datafication and automation in IVF (Cohen 2013; Sterckx,et al. 2017; Van de Wiel 2019). Critics have advocated for stronger regulation of add-ons (Hendriks and Pearson 2018; Howard, 2018; Rutherford 2017) as globally the requirements for introducing treatments into practice are relatively limited; for example, they do not include demonstration of effectiveness and safety in randomized controlled trials (RCTs). A particular concern is that, without performing high-quality trials, it cannot be known whether an add-on causes unanticipated harms or actually worsens treatment outcomes.

In the UK, the HFEA has set up a traffic light system to provide independent information on the current state of evidence for add-ons (HFEA, 2018). A red light means there is no evidence to show that the add-on is effective and safe. Amber means there is a small or conflicting body of evidence, which means further research is still required and the technique cannot be recommended for routine use. A green light is given when more than one good-quality RCT shows that the procedure is effective and safe. Currently, there are no add-ons with a green rating. Artificial egg activation, elective freeze-all cycles, embryo glue, endometrial scratching and time-lapse imaging (TLI) are currently rated amber, while assisted hatching, intrauterine culture, preimplantation genetic testing for aneuploidies (PGT-A), reproductive immunology, intracytoplasmic morphologic sperm injection and physiological intracytoplasmic sperm injection are rated red. The HFEA is in the process of expanding the add-on list.

Globally, data on add-on use are limited but are expected to vary according to the source of funding. For example, in the Netherlands, where treatment is largely publicly funded, add-ons are relatively uncommon. In countries with a greater proportion of privately funded treatment, the use of add-ons is anticipated to be greater. An example is the UK, where over 60% of IVF treatments are self-funded and add-on treatments typically come with an additional price tag for the patient (Rutherford, 2017). A 2016 review of UK fertility clinic websites suggested that add-ons were commonly advertised, and that claims of benefit were usually not accompanied by references to published studies (Spencer et al., 2016). However, that review was not specifically targeted at add-ons, and included some medically necessary treatments (e.g. surgical sperm retrieval used for severe male factor infertility). There is a clear need for more data on the provision and presentation of add-ons so that ongoing discussions can be conducted with recourse to the facts.

To this end, this study reviewed the prevalence, pricing and promotion of three of the main add-ons: TLI, PGT-A and assisted hatching.

## Materials and methods

### Data collection and analysis

The HFEA ‘Choose a fertility clinic’ website service (HFEA, 2020) was used to identify UK IVF clinics and their websites. Only clinics that offered IVF treatment were considered. ‘Satellite’ clinics or small ‘treatment-only’ clinics offering only intrauterine insemination, or fertility assessments and diagnostics, were not eligible, nor were gamete biobanks.

A search was made for information on and advertising of assisted hatching, TLI and PGT-A on the eligible websites in May 2019. For each clinic, a record was made of whether each of the add-ons was advertised. If the clinic advertised the adjunct, screenshots were taken of the webpages, including the claims made in relation to the add-ons. The price of the procedure was also recorded at this stage. Next, the claims text was analysed by categorizing each claim according to its content and the particular language used. To give concrete examples, instances were recorded of where a website claimed an add-on improved implantation chances or rates, or improved IVF success rates, or stated that insufficient evidence was available. As many claims were recorded for each website as were made. Advertisements were identified by a single reviewer. Categorization of claims was double-checked by a second reviewer.

Statistical analyses were restricted to descriptive analyses of the frequency and pricing of each add-on, and the frequency of claims made in relation to their use. The analysis was conducted at the level of individual clinics, but recorded cases where clinics were members of larger groups.

## Results

**Figure 1:**
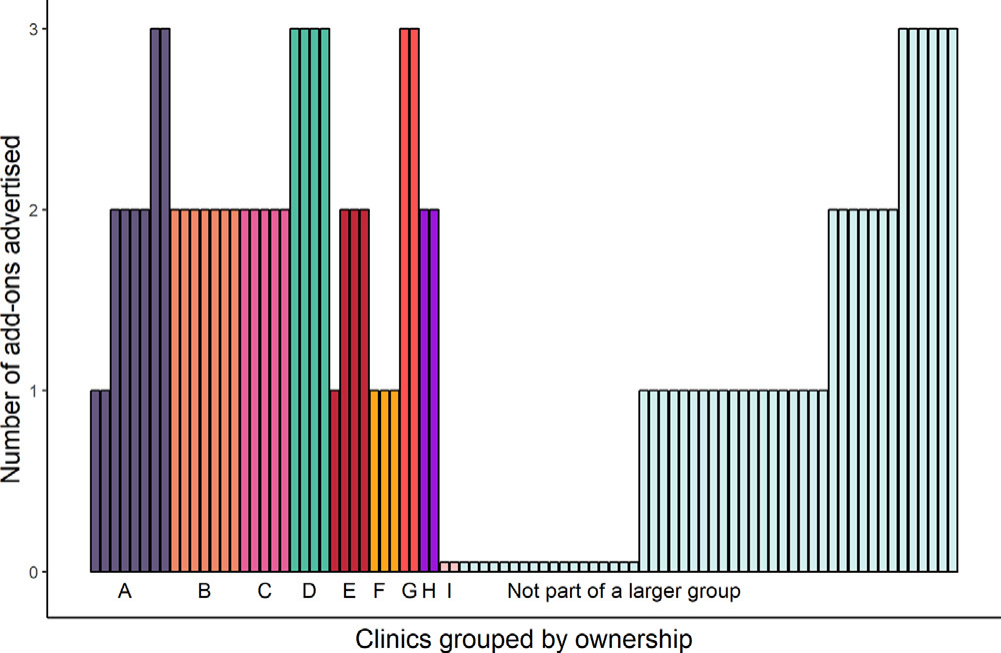
Number of add-ons advertised by each clinic, out of the three add-ons investigated. A single bar represents one clinic. Clinics are arranged according to ownership group (labelled A to I), denoted by colour.

A total of 87 different clinics, with 72 unique websites, was identified; 37 (43%) clinics were part of one of nine groups of IVF clinics, of sizes ranging from two to eight clinics in the UK (Figure 1).

Sixty-seven (77%) clinics advertised one or more of the three add-ons, with a median (interquartile range) of 1 (1–2). The number of add-ons advertised was not always consistent across clinics within a group of clinics (Figure 1).

**Figure 2:**
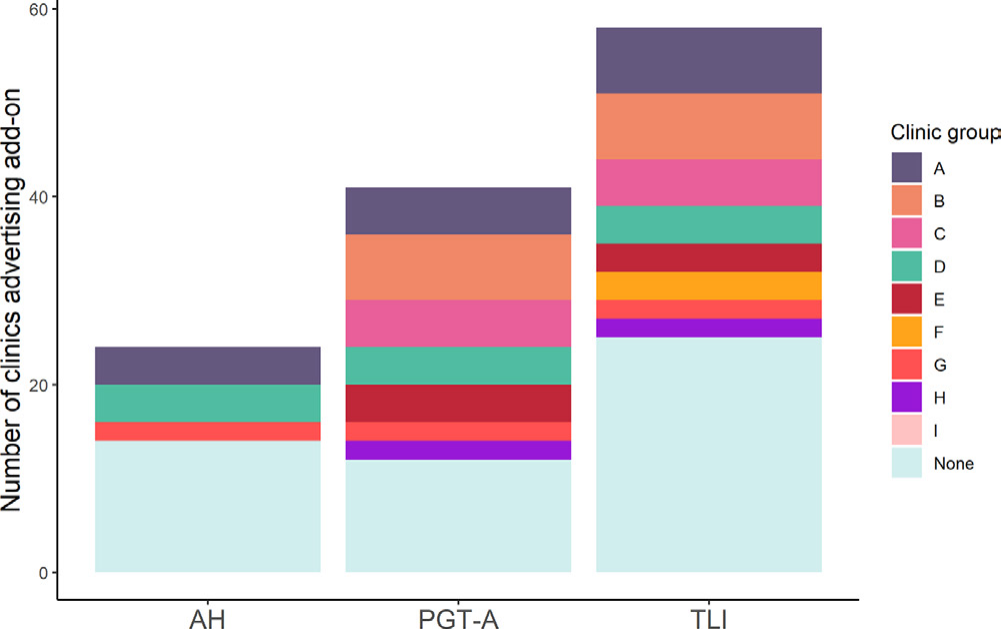
Number of clinics advertising each add-on, coloured according to ownership group. AH, assisted hatching; PGT-A, preimplantation genetic testing for aneuploidies; TLI, time-lapse imaging.

TLI was the most frequently advertised of the three add-ons (67%), followed by PGT-A (47%) and then assisted hatching (28%) (Table 1, Figure 2). TLI and PGT-A were most frequently advertised as stand-alone products, but were available as part of a package at some clinics (Table 1).

**Table 1:** Advertisement and pricing of add-ons on clinic websites

Add-on	Number (%) advertising	Price (£) Median, interquartile range, range
Assisted hatching	24 (28)	450, 288–481, 130–600^a^
PGT-A	41 (47)	
Stand-alone	36 (41)	2695, 2500–2850, 2100–3295^b^
As part of package	5 (6)	9500, 6460–9500, 4230–9500
Time-lapse embryo imaging	58 (67)	
Stand-alone	47 (54)	478, 300–699, 0–795^c^
As part of package	11 (13)	4020, 3608–4638, 2950–6975
Number of add-ons advertised		
0	20 (23)	
1	25 (29)	
2	28 (32)	
3	14 (16)	
Median (IQR)	1 (1–2)	

a Not reported for four clinics.

b Not reported for three clinics.

c Not reported for five clinics. IQR, interquartile range; PGT-A, preimplantation genetic testing.

### Claims made about add-ons

Table 2 summarizes the claims made in relation to the add-ons on clinic websites. Very few websites stated that the effectiveness of the add-on was in doubt or unclear: one website stated this for assisted hatching, four stated this for TLI, and two stated this for PGT-A. No website acknowledged that there could be a negative impact of an add-on (e.g. a reduction in live birth rate). Most claims of efficacy were based on outcomes such as implantation or pregnancy rates rather than live birth; nine (assisted hatching), 12 (TLI) and five (PGT-A) clinics claimed an improved implantation rate associated with the add-on, while five (assisted hatching), 21 (TLI) and 15 (PGT-A) claimed an improvement in pregnancy rates.

**Table 2:** Claims made in relation to three add-ons on IVF clinic websites

Type of advertisement	Number of websites
Assisted hatching	
Improves implantation chances/rates	9
Improves pregnancy/clinical pregnancy chances/rates	5
Evidence-based studies	7
Insufficient/no evidence	1
Reference to possible negative impact (e.g. on live birth)	0
Time-lapse embryo imaging	
Improves IVF success rates	11
Improves clinical outcomes	3
Improves implantation chances/rates	12
Improves/increases ongoing pregnancy chances/rates	21
Evidence-based studies/research/RCT	22
Reference of studies	3
Insufficient/no evidence	4
Improves embryo selection – selection of ‘highest potential’ embryo	30
Improves embryo culture and manipulation conditions	8
Significant reduction of miscarriage/early pregnancy loss rates	10
Higher percentage of genetically normal blastocysts – improves embryo potential	8
Increases live birth rate	3
Reduces preterm birth and very low birth weight	1
Improves birth rates	1
Supports better embryo development	11
Reference to possible negative impact (e.g. on live birth)	0
PGT-A	
Improves pregnancy rates/likelihood	15
Improves live birth rates	5
Improves implantation rates	5
Evidence-based studies/research	7
Reference of studies	2
Improves IVF success rates	4
Reduces chance of miscarriage/minimizes chance of poor outcome	10
Increases chance of having a healthy baby	3
Does not increase overall chance of having a baby/no guarantee of a pregnancy	2
Reference to possible negative impact (e.g. on live birth)	0

PGT-A, preimplantation genetic testing for aneuploidies; RCT, randomized controlled trial.

Some clinics suggested that the add-on would improve live birth rates; four clinics claimed that TLI would improve ‘live birth’ or ‘birth’ rates, while eight claimed that PGT-A improved live birth rates or the chance of having a healthy baby. No clinic claimed that assisted hatching would improve live birth rates.

### Pricing

Median prices were £450 for assisted hatching, £478 for TLI and £2695 for PGT-A when the add-ons were priced as stand-alone items. There was substantial variation in pricing for each add-on (Table 1). The price for assisted hatching ranged from £130 to £600.^.^ Prices for TLI as a stand-alone add-on ranged from £0 to £795. Four clinics (all unaffiliated to a larger group) did not charge for TLI, while the most expensive 25% charged £699 or higher. The difference in pricing for stand-alone PGT-A was nearly £1200 between the lowest (£2100) and highest (£3295) prices. Packages including TLI or PGT-A also varied substantially in price, with more than a two-fold difference between the lowest and highest priced packages, although it is important to note that these packages probably varied in relation to a number of different aspects.

## Discussion

The present review provides evidence that add-ons are commonly offered in the context of self-funded treatment, and that they are frequently marketed using claims that are not clearly supported by robust evidence. The review also found pricing to be variable, and established that almost half (43%) of UK IVF clinics are part of larger groups.

A key implication of these results is that the promotion and provision of add-on treatments with a limited evidence base is common. As advertisements were identified by a single reviewer, it is plausible that some instances of an add-on being advertised could have been missed. This would make the estimates of the prevalence of these add-ons conservative. There is ongoing debate over the ethics of providing infertility treatments that have not been proven to be efficacious and safe (e.g. Hendriks and Pearson, 2018; Macklon et al., 2019; Repping, 2019). One argument is that the provision of unproven treatments is ethical provided that sufficient information is given to allow informed consent. In order to make an informed decision in this regard, it is essential that the current state of knowledge about a treatment's effectiveness and safety is transparently conveyed to the patient. The study's findings, however, show that prospective patients encounter marketing claims on clinic websites that do not clearly portray the scientific uncertainties around these treatments and are strongly, perhaps misleadingly, suggestive of benefit.

Interpreting complex clinical evidence can be challenging for all stakeholders, and variation in the measures and terms used to describe performance of treatments has previously been identified as a potential source of confusion (Goodman et al., 2020; Wilkinson et al., 2017). In general, the IVF community are in agreement that the primary outcome for studies looking at the effectiveness of treatments is live birth rate (Legro et al., 2014) and this is the criterion that the HFEA have adopted for their traffic light system. This study has shown that the language clinics use to advertise add-ons does not reflect this consensus and is not consistent, including phrases such as ‘improves IVF success’, ‘improves implantation rates’, ‘improves embryo selection’ and ‘improves the chance of a healthy baby’. It may not always be clear to prospective patients that improvements in upstream outcomes such as embryo quality, implantation or even pregnancy frequently do not translate into improved live birth rate.

Where a treatment has not been robustly evaluated, there remains the possibility that it could actually reduce the chances of having a healthy baby, or otherwise cause harm; an example of the former is first-generation preimplantation genetic testing (Twisk et al., 2006). If patients are to make an informed decision about unproven treatments, they should be made aware that some add-ons might plausibly reduce their chances of having a baby. Few websites mentioned that benefits were uncertain, and none mentioned the possibility of a reduced live birth rate or other potential negative effects.

These points, together with the variation in pricing observed in the present study, suggest that there may at times be a tension between the direct-to-consumer marketing of add-ons as revenue-generating products and transparent disclosure about their efficacy, safety and supporting evidence base. It is important to distinguish marketing claims from clinical counselling provided on attendance at the clinic, however, as the present study reveals nothing about direct clinician–patient interactions. It is to be expected that more nuanced discussions around the suitability of treatments, incorporating possible benefits, risks and individual values, take place during the patient consultation, although this is an area that would benefit from more data. It is unclear, however, whether this opportunity to subsequently revise the claims made on clinic websites alleviates concerns over patients being potentially insufficiently or incorrectly informed to begin with. Moreover, clinicians may cite the enthusiasm of a patient to try a particular add-on as part of the reason for providing it, raising questions about the ethics of websites making enthusiastic claims in the first place.

While data specifically relating to the marketing of add-ons have been limited prior to this study, the current findings are in line with previous reviews looking at the quality of information about infertility on patient-facing websites. In the USA, several reviews have raised concerns over transparency of information on clinic websites (Huang 2005; Klitzman et al., 2009) or poor adherence to societal guidelines (Abusief et al., 2007; Hawkins 2013). Meanwhile, a recent review of clinic websites in Australia and New Zealand has highlighted the fact that adherence to reporting guidelines does not necessarily guarantee a high standard of information for patients (Goodman et al., 2020). Other authors have pointed out the challenges of assessing the reliability of IVF clinic websites as sources of health information, given their function as marketing tools (Jain and Barbieri, 2005).

There have been calls for more regulation and RCTs of add-ons in response to concerns about the pressures to provide a return on investment to shareholders and investors (Repping, 2019). This is particularly pertinent given that, in the last decade, the IVF sector has attracted an increasing amount of venture capital and private equity investment and has experienced a consolidating trend characterized by mergers of clinics into bigger fertility groups and acquisitions of clinics and biotechnology companies by larger enterprises (Van de Wiel, 2020; Williams et al., 2017). In the UK, the presence of independent IVF clinics has reduced as chains of clinics are established. This study shows for the first time that 37 out of 87 clinics (43%) are part of one of nine groups ranging in size from two to eight clinics in the UK. Clinics within a group did not necessarily offer the same IVF add-ons. For example, in the largest group with eight clinics, two clinics advertised one add-on, four advertised two add-ons, and two advertised all three add-ons examined in this study. In order to understand why there are differences, the current authors are undertaking a study to interview the medical directors of IVF clinics to examine this further.

This study has shown that there is substantial variation in the price clinics charge for add-ons. Two of the IVF add-ons in this study, assisted hatching and PGT-A, are rated red by the HFEA as there is currently no evidence to show that they improve live birth rates – in the case of PGT-A, there is some good evidence to the contrary (Munné et al., 2019). It should be noted that, at the time of data collection for this study, PGT-A had an ‘amber’ rating, which was subsequently changed to red after taking into account the results from the European Society of Human Reproduction and Embryology (ESHRE) (Single Embryo TrAnsfeR of Euploid Embryo) (Munné et al., 2019; Verpoest et al., 2018). Nevertheless, 28% of clinics advertise assisted hatching and 47% advertise PGT-A, and none offers the treatments free of charge. The charge for assisted hatching was on average about £450. The median price for PGT-A was £2695 but the difference in pricing was nearly £1200 between the lowest and highest prices. Four clinics did not charge for TLI, but the cost reached £699 or higher in 25% of clinics.

How much do add-ons actually cost the clinic? This question is difficult to answer. For example, assisted hatching will take up a small amount of additional time for the embryologist to perform the procedure, but also requires laboratory consumables and equipment. Similarly, the cost of TLI includes the cost of purchase or lease of the incubator. The climate of preimplantation genetic testing has changed dramatically over the last 25 years from small laboratories, many offering in-house services in collaboration with a single IVF clinic, to companies offering their genetic services to multiple IVF clinics, often across borders. One reason for this has been the expensive equipment required to undertake preimplantation genetic testing, and another the reduced cost when samples are batched. This has been possible using of vitrification so that embryos can be biopsied and vitrified, and the samples sent anywhere in the world for their genetic diagnosis with none of the time restrictions that used to occur when a fresh embryo transfer was required.

In order to produce the best possible information about the effectiveness and safety of treatment, it is essential that clinics are encouraged to take part in high-quality RCTs, and that barriers to conducting responsible trials are reduced. The principle is simple; if a treatment works, it can be shown to work in good RCTs. Moreover, if there are reasons to believe that an intervention should work in a particular patient group, trials should be conducted in that group. By following this principle, it will be possible to distinguish effective add-ons from those that are deleterious or neutral. It is important to note that even neutral add-ons have associated harms, as money spent on an ineffective add-on cannot be spent on an effective alternative (such as a further IVF cycle, where this is clinically appropriate). The expectation would then be that effective add-ons might be absorbed into routine care, although this is likely to depend on cost. Until this becomes the routine pathway for the introduction of new fertility treatments, we should not be surprised if some add-on treatments turn out to result in more harm than good.
